# Mass cytometry analysis of blood from peanut-sensitized tolerant and clinically allergic infants

**DOI:** 10.1038/s41597-022-01861-x

**Published:** 2022-12-01

**Authors:** Amanda R. Tursi, Nicholas K. Saba, Diane Dunham, Monali Manohar, Rachel L. Peters, Richard Saffery, Jennifer J. Koplin, Kari C. Nadeau, Melanie R. Neeland, Sandra Andorf

**Affiliations:** 1 Division of Biomedical Informatics, Cincinnati Children’s Hospital Medical Center, Cincinnati, OH USA; 2 Department of Biomedical Informatics, University of Cincinnati College of Medicine, Cincinnati, OH USA; 3 Sean N. Parker Center for Allergy & Asthma Research, Stanford University, Stanford, CA USA; 4 Murdoch Children’s Research Institute, Royal Children’s Hospital, Parkville, VIC Australia; 5 Department of Paediatrics, The University of Melbourne, Parkville, VIC Australia; 6 Department of Pediatrics, University of Cincinnati College of Medicine, Cincinnati, OH USA; 7 Divisions of Allergy and Immunology and of Biostatistics and Epidemiology, Cincinnati Children’s Hospital Medical Center, Cincinnati, OH USA

**Keywords:** Immunological disorders, Immunology

## Abstract

IgE-mediated food allergies in infants are a significant health concern, with peanut allergy being of particular interest due to its prevalence and severity. Among individuals who produce peanut-specific IgE some experience no adverse reaction on peanut consumption. This asymptomatic phenotype is known as sensitized tolerance. To elucidate the immune environment of peanut sensitized tolerant and clinically allergic one-year-olds, high-dimensional mass cytometry was conducted as part of the HealthNuts study. The resulting data includes peripheral blood mononuclear cells from 36 participants encompassing non-allergic, peanut sensitized with tolerance, and clinically peanut allergic infants. The raw mass cytometry data is described here and freely available for reuse through the Immunology Database and Analysis Portal (ImmPort). Additional allergy information and serum vitamin D levels of the participants were measured and are also included in the data upload. These high-dimensional mass cytometry data, when combined with clinical information, offer a broad immune profile of peanut allergic and sensitized tolerant infants.

## Abstract (summary)

**Table:** 

Measurement(s)	expression profiling
Technology Type(s)	cytometry time of flight assay
Sample Characteristic - Organism	Homo sapiens
Sample Characteristic - Location	Australia

## Background & Summary

The prevalence of pediatric food allergy is increasing, constituting a significant health concern worldwide^1^. Already, global studies estimate 5–10% of young children are food allergic, with peanuts being one of the most common allergens^1–3^. These food allergies often place a high burden on the affected child, their family, and various social institutions. Allergy symptoms can be severe and life-threatening, with 42% of food allergic children having at least one emergency department visit associated with their condition during childhood^3^. In particular, peanut allergies have increased prevalence, severity, and are less likely to naturally resolve compared to other types of food allergies^3–5^. Food allergies are also associated with impeded social dynamics and increased anxiety in both allergic children and their caregivers^6–9^. Negative impacts reach beyond the child and their family, as food allergies have also become a significant burden on schools, day cares, and the overall healthcare system^10,11^.

Skin prick testing (SPT) and the measurement of allergen-specific IgE (sIgE) are routinely performed to evaluate food sensitization. However, sensitization does not necessarily equate to clinical allergy and some individuals who produce sIgE can consume the food without an adverse reaction^12^. This phenomenon, known as sensitized tolerance, is an added complexity in the mechanisms that underlay IgE-mediated food allergies. Although some studies have sought to characterize peanut sensitized tolerant, sensitized and clinically allergic, and non-sensitized children, much of the immune response is still unknown^13–15^. Of particular interest are infants during the first year of life, who are undergoing immune system development and with whom allergy prevention may still be possible^16–18^.

The main study connected to this data descriptor aimed to characterize the immune system in peanut sensitized tolerant and clinically peanut allergic one-year-old infants^19^. High-dimensional mass cytometry was used to analyze samples from a subset of infants from the HealthNuts study^20^. HealthNuts is an ongoing population-based longitudinal study focused on allergies in Australian children. Our cohort consisted of three groups (n = 12 each): infants with peanut sensitization with tolerance (PST), those that were clinically considered peanut allergic (PA), and non-sensitized, non-peanut allergic infants (NA). Groups were determined based on SPT and sIgE levels, in addition to peanut oral food challenges (OFC). OFCs are the gold standard to determine a clinical food allergy^21,22^. Peripheral blood mononuclear cells (PBMC) were left in culture unstimulated, stimulated with peanut protein solution for 24 hours, or stimulated with phorbal myristate acetate (PMA) and ionomycin for 4 hours prior to being assessed by mass cytometry.

Analyses of unsupervised clustering results using FlowSOM, after normalization with the landmark alignment procedure, revealed PST to be associated with increased frequency of plasmacytoid DCs and decreased frequency of naïve CD4 T cells^19,23,24^. PA was associated with a higher occurrence of activated B cells (CD19^hi^HLADR^hi^). Functional analysis revealed increased global expression of TNFα across all single cells in PA infants. Additionally, IL-2 expression was increased in the naïve CD4 T cell cluster in PST infants. An examination of the peanut-specific T cell response by expression of CD40L and CD69 on CD4 T cells following peanut stimulation revealed that PA is characterized by increases in peanut-activated CD4 T cells that display a memory phenotype. This trend was not observed in the PST infants.

Additional research utilized a subset of mass cytometry samples from the initial study to explore the relationship between serum vitamin D (25(OH)D_**3**_) concentration and peanut and/or egg allergy in infants^25^. The findings of this analysis suggest that serum vitamin D enhances circulating levels of Tregs in food-allergic infants.

As the vitamin D study illustrates, there is reuse potential in this mass cytometry profiling of one-year-olds with food sensitization, food allergies, and healthy controls. There is limited publicly available cytometry data concerning allergies in such a young cohort of subjects. This data is part of the HealthNuts study, which is well-characterized and contains high-quality demographic and phenotypic information. Food allergic/sensitization-related information include sIgE and outcomes of OFCs to peanut, egg, and sesame. The dataset also contains SPT results for peanut, egg, sesame, milk, and house dust mite. Consequently, the data can be used for hypothesis generation related to food allergy and sensitization in infants. Additionally, methods to conduct meta-analysis of cytometry data have been created, offering further potential for reuse^26,27^. The original, ungated fcs files and corresponding participant information have been uploaded to the Immunology Database and Analysis Portal (ImmPort) for public access^28–31^.

## Methods

The following methods are expanded versions of descriptions in our related work^19,25^.

### Study participants and phenotype measurements

A subset of participants (n = 36) from the HealthNuts study (n = 5276) were selected for this study^19,20^. The infants selected for mass cytometry analysis were chosen to ensure equal numbers (n = 12) of challenge-confirmed peanut-allergic infants (PA), peanut-sensitized tolerant infants (PST), and non-sensitized, non-food-allergic (NA) infants. Peanut SPT was measured for all participants and peanut sIgE for a subset (n = 30). All participants in our study underwent an OFC to peanut according to standardized protocols^32^. The food challenge was conducted at clinic appointments with a gradual increase of doses on day 1, with the maximum day 1 dose being 1 teaspoon of peanut butter (cumulative dose 2 teaspoons). The OFC continued at home for days 2 through 7, with ingestion of the day 1 maximum dose. The OFC was terminated and considered positive if any of the following occurred within 2 hours of ingestion: 3 or more concurrent noncontact urticaria persisting for at least 5 minutes; perioral or periorbital angioedema; vomiting; or evidence of circulatory or respiratory compromise.

Although infants were chosen for mass cytometry analysis based on results of a peanut OFC, the same infants also underwent SPT and OFC for eggs and sesame. The maximum day 1 dose for eggs was set as 1 whole raw egg white (cumulative dose 1 whole raw egg white). For sesame, the maximum dose was 1 teaspoon of tahini paste (cumulative dose 9.7 ml of tahini). SPTs, but not OFC, were also performed for cow’s milk and house dust mite. Additionally, sIgE levels to peanut (n = 30), egg (n = 30) and sesame (n = 31) were recorded for the majority of participants. Serum sIgE was measured using the ImmunoCAP System FEIA (Phadia AB).

Infants were assigned to the PA group if they had a SPT wheal diameter ≥2 mm or a peanut sIgE level of ≥0.35 kUA/L and had a positive OFC to peanut at age 1 year. Infants in the PST group had a peanut SPT wheal diameter ≥2 mm and peanut sIgE level of ≥0.35 kUA/L, together with a negative peanut OFC at age 1 year. The infants in the NA group were non-sensitized (negative SPT to peanut, egg, sesame, and cow’s milk) and had a negative peanut OFC outcome at age 1 year. Compared to the threshold of ≥3 mm often used in clinical practice, a lower SPT wheal diameter threshold of 2 mm or larger was used to determine sensitization at age 1 year, as there is evidence that infants aged 12 months may have lower skin reactivity than older children^33^. However, the peanut SPT results of all participants are shared as part of this dataset so that a different threshold can be utilized in secondary analyses of these data.

Additional information that was recorded and is shared with this data description include: current eczema status of participants, family history of allergy, any siblings with allergy, consumption of allergenic foods (any peanut or egg), and ancestry information (parent country of birth). A family history of allergy was based on responses to the question “does anyone in your family suffer asthma, eczema, hay fever, food allergy (please specify food)”^34^. Current eczema was measured at study recruitment by clinical staff^35^. Nurses were instructed to examine patients for hallmark signs of eczema, including erythema, excoriation, oedema/papulation, lichenification, and vesiculation. Clinical staff were referred to online SCORAD training photo tutorials to use as a reference for the appearance of eczema. Any presence of eczema, no matter the severity, was considered positive for the ‘current eczema’ criteria.

Serum concentration of vitamin D in the form of serum 25-hydroxyvitamin D_**3**_ (25(OH)D_**3**_) was measured by liquid chromatography tandem mass spectrometry as previously described^36,37^. Briefly, extracts were derivatized with 4-phenyl-1,2,4-triazoline-3,5-dione prior to analysis with liquid chromatography tandem mass spectrometry. A sinusoidal curve was fitted to data on 25(OH)D_**3**_ levels and the date blood was sampled, with the difference between observed and fitted vitamin D levels taken (i.e. the regression residual) and added to the population’s average vitamin D level. This was done to represent a seasonally adjusted measure of vitamin D.

### Cell culture and preparation of cells for mass cytometry

Up to 7 ml of blood was collected at clinic appointments within 2 hours of the final dose of an OFC. The blood was collected into a sodium heparin tube (Sarstedt). PBMCs were separated from whole blood by density gradient centrifugation within 1 hour of blood collection. First, plasma was isolated by centrifugation at 700 × g for 10 minutes at room temperature. RPMI media was added to cells in a 1:1 ratio before layering onto 5.0 ml of Ficoll-Paque solution. Brake-free centrifugation was done at 400 × g for 30 minutes and PBMCs at the interface were aspirated and washed in RPMI containing 2% heat-inactivated fetal calf serum. PBMCs were centrifuged at 500 × g for 7 minutes and cryopreserved in liquid nitrogen at 10 × 10^6^/ml in RPMI with 15% dimethyl sulfoxide in fetal calf serum.

Cell culture media consisted of RPMI supplemented with 10% heat-inactivated fetal calf serum and penicillin streptomycin. PBMCs were thawed in 10 ml of the media containing 25 U/ml benzonase at 37 °C and centrifuged at 300 × g for 10 min. Following this, cells were washed twice in culture media. The NucleoCounter NC-200 was used for viability count and the mean viability across all samples was found to be 90.5%. For overnight rest, cells were resuspended at 2 × 10^6^/ml in cell culture media in a T25 flask at 37 °C and 5% CO2. The next day, cells were resuspended at 3 × 10^6^/200 µL. They were then cultured in U-bottom 96-well plates at 200 µL/well with (i) media alone for 24 hours, (ii) 200 µg/ml of endotoxin cleaned pure peanut protein solution (Greer: XPF171D3A2.5: Ara h 1 content: 71.03 µg/ml, Ara h 2 content: 78.43 µg/ml) for 24 hours or (iii) 20 ng/ml PMA and 1 µg/ml ionomycin combined solution for the concluding 4 hours.

To ensure the cells were responsive to stimulation, PMA/ionomycin was used as a positive control and a non-specific cell stimulus in the assay. Brefeldin-A was added to all wells after 20 hours to inhibit extracellular cytokine transport. After cell culture, PBMCs were centrifuged at 300 × g for 7 minutes before being resuspended in 200 µl-filtered CyFACS buffer (0.1% bovine serum albumin, 0.1% sodium azide, 2 mM EDTA in PBS).

Prior to barcoding, all cell staining steps were performed in V-bottom 96-well plates. The staining included wash steps in 200 µl CyFACS buffer and centrifugation at 300 × g for 7 minutes. PBMCs were resuspended in 70 µl of our surface antibody cocktail (Table 1) prior to being incubated for 30 min at room temperature. Cells were washed three times prior to resuspension in 100 µl of live/dead 115-DOTA maleimide (stock 5 mg/ml, diluted 1:3000) for 15 minutes at room temperature. Cells were washed an additional three times before transferring into polypropylene fluorescence-activated cell sorting tubes. Barcoding was performed using the Cell-ID 20-Plex Pd Barcoding Kit (Fluidigm) according to manufacturer’s instructions. PBMCs were resuspended in 100 µl of 2% paraformaldehyde (PFA) in CyPBS (filtered PBS) overnight at 4 °C.

**Table 1:** Mass cytometry antibody panel.

Metal label	Surface Marker	Manufacturer	Clone	Vol (µl)/70 µl reaction
115In	live/dead			
113In	CD86	BD, conjugated in-house	IT2.2	1.4
142Nd	CD19	Fluidigm	HIB19	0.7
143Nd	CD49b	Biolegend, conjugated in-house	P1E6-C5	1.4
145Nd	CD4	Fluidigm	RPA-T4	0.7
146Nd	CD8	Fluidigm	RPA-T8	0.7
147Sm	CD20	Fluidigm	2H7	0.7
148Nd	CD38	BD, conjugated in-house	HB-7	0.7
149Sm	CCR4	Fluidigm	205410	0.7
150Nd	LAG3	Fluidigm	874501	0.7
151Eu	CD123	Fluidigm	6H6	0.7
153Eu	CD45RA	Fluidigm	HI100	0.7
154Sm	CD3	Fluidigm	UCHT1	0.7
155Gd	CD28	BD, conjugated in-house	L283	0.7
157Gd	HLA-DR	BD, conjugated in-house	G46-6	0.7
158Gd	CD33	Fluidigm	WM53	0.7
159Tb	CD11c	Fluidigm	Bu15	0.7
160Gd	CD14	Fluidigm	M5E2	0.7
163Dy	CXCR3	Fluidigm	G025H7	0.7
165Ho	CD127	Fluidigm	A019D5	0.7
167Er	CD27	Fluidigm	L128	0.7
169Tm	CCR7	R&D Systems, conjugated in-house	15053	1.1
173Yb	CD25	BD, conjugated in-house	M-A251	0.7
176Yb	CD56	Fluidigm	NCAM16.2	0.7
209Bi	CD16	Fluidigm	3G8	0.7
**Metal label**	**Intracellular Marker**	**Manufacturer**	**Clone**	**Vol (µl)/70 µl reaction**
144Nd	IL-4	Fluidigm	MP4-25D2	0.7
152Sm	TNFα	Fluidigm	Mab11	0.7
161Dy	IFNγ	eBioscience, conjugated in-house	4 S.B4	0.7
162Dy	CD69	Fluidigm	FN50	0.7
164Dy	IL-17	Fluidigm	N49-853	0.7
166Er	IL-2	Fluidigm	MQ1-17h12	0.7
168Er	CD40L	Fluidigm	24-31	0.7
171Yb	IL-10	Biolegend, conjugated in-house	JES3-9D7	0.7

Cells were then resuspended in 2 ml CyFACS buffer and centrifuged at 600 × g for 5 minutes at 4 °C. Cell count was performed to ensure that an equal number of cells from each infant were used to pool into a single 15 ml tube. The tube was then centrifuged at 600 × g for 5 minutes at 4 °C. To achieve permeabilization, cells were resuspended in 2 ml of permeabilization buffer (EBioscience) and centrifuged at 600 × g for 5 minutes at 4 °C. A second wash in 2 ml permeabilization buffer was performed. Next, the pooled cells were resuspended in 100 µl of intracellular antibody cocktail (Table 1) and incubated for 30 minutes at room temperature. Cells were washed in 2 ml of permeabilization buffer, followed by two washes in 2 ml CyFACS buffer. For each sample, 100 µl of Ir-Interchelator (1:2000, diluted in 2% PFA in CyPBS) was added. All samples were incubated overnight at 4 °C.

Cells were washed again on the day of mass cytometry acquisition. All washes were in 2 ml volumes and centrifugation was done at 600 × g for 5 min at 4 °C. CyFACS buffer was used for two washes, followed by one wash in CyPBS, and two additional washes in milliQ water. The Helios mass cytometer from Fluidigm was used for single-cell detection analysis and detection. Standard instrument setup procedures were followed.

FCS files gated for live single cells were used for the computational analysis and manual gating conducted for both previously published studies that use this data^19,25^. The gating strategy is illustrated in Figure S6 from the primary study connected with this dataset^19^.

### Ethics

The HealthNuts study has ethics approval from the Victorian State Government Office for Children (CDF/07/492), the Victorian State Government Department of Human Services (10/07), and the Royal Children’s Hospital Human Research Ethics Committee (27047). Informed consent was obtained from parents or guardians of all participants, and participants and families have consented to information sharing.

## Data Records

All raw FCS files are accessible through ImmPort (study accession: SDY2015)^31^. The deposited files consist of samples from thirty-six unique individuals, split into three groups: PA, PST, and NA. Each group is comprised of data from twelve one-year-olds.

For each individual, PBMCs underwent three forms of stimulation. One subset was stimulated with peanut solution (allergen-specific stimulus), while another group was stimulated with PMA and ionomycin (non-specific stimulus). The third condition was media alone (no stimulus). One individual (P33) did not have cells stimulated with the peanut solution. In total, 107 raw FCS are available for open access.

**Fig. 1:**
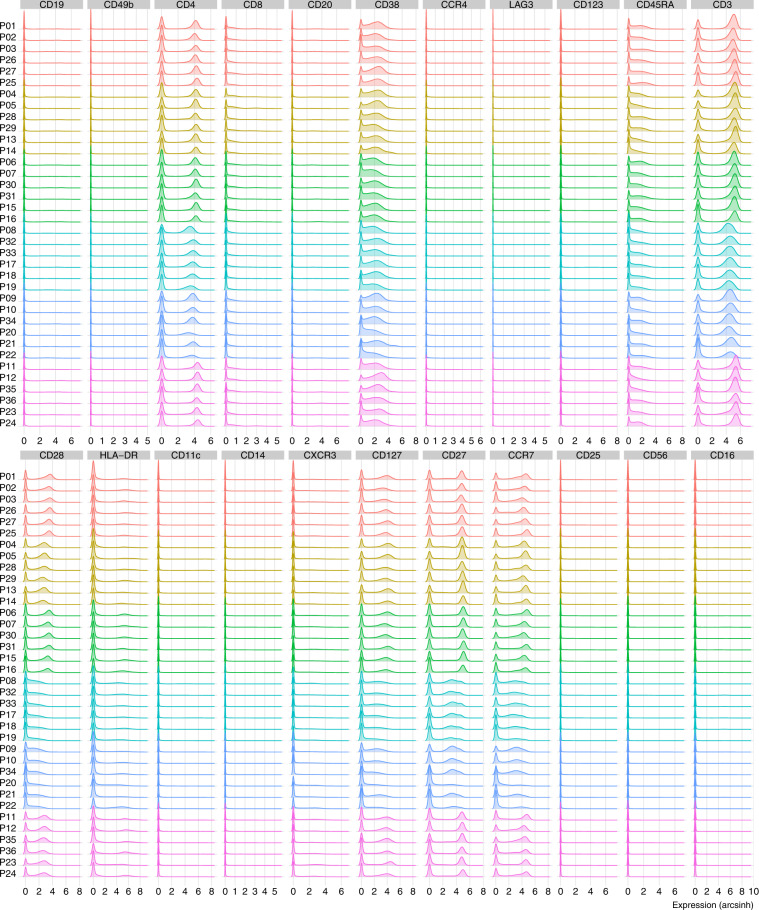
Densities of the arcsinh-transformed expression values of 22 surface markers in the unstimulated samples (PBMCs). Each row represents one FCS file, corresponding to one of the 36 infants from the HealthNuts study, and each column specifies the protein marker. For each file, 100,000 live, single cells were randomly selected for plotting. The data were acquired in 6 batches, color-coded from top (batch 1) to bottom (batch 6).

A panel of antibodies against 32 markers was used in the design of this experiment (Table 1). CD86 and CD33 were excluded from any analysis in the original studies due to technical concerns. CD86 stocks depleted midway through sample processing and so was only added in samples that were included in batches 1–3. The use of CD33 displayed irregular results due to a technical issue associated with the metal tag. The expression densities of the surface markers in the live, single cells of the unstimulated samples are depicted in Fig. 1.

In addition to the raw FCS files, information about the samples and participants are included in our ImmPort upload. In the ImmPort data model (https://www.immport.org/shared/dataModel), information about OFC, SPT, family history, and questionnaire were included in the *Assessment Panel* and *Assessment Component* tables. sIgE and vitamin D measurements were uploaded into the *Lab Test Panel* and *Lab Test* tables. The batch information for each experimental sample, and thus each FCS file, was provided in the *description* column of the *ExpSample* table. For access to the sample and participant data without the need to know the ImmPort data model and the requirement to query for the data across several tables, the information was also summarized in two tables that were uploaded to ImmPort and listed in the *Study File* table. Descriptions of each FCS file, including batch information, are in HN_CyTOF_fcsFiles.csv. Patient demographic data and allergy-related information (including OFC, SPT, and sIgE) are in HN_CyTOF_Participants.csv. In both files, the first column contains the subject accessions assigned by ImmPort, while the second column shows the participant IDs as used in the manuscripts utilizing these data. The mapping between ImmPort assigned subject accessions and the participant IDs used in our work is also provided in the *Subject* table in the data model in which our assigned IDs were provided in the *description* column and the ImmPort IDs are in the *subject_accession* column.

## Technical Validation

The duration for *ex vivo* peanut stimulation was based on efficient detection of peanut-specific (CD69^+^ CD40L^+^) CD4+ T cells through time course (8, 24, 48, 72 hour-long stimulation) experiments. The successful stimulation of peanut-specific T cells by the peanut protein solution was shown in the work related to this dataset (refer to Figures 5, S5 and the corresponding Source Data file in^19^). Stimulation with PMA/ionomycin resulted in a significant increase in the percentage of TNFα, IFNγ and IL-2 producing CD4+ and CD8+ T cells compared to unstimulated cells (Supplementary Figure S1), validating this stimulation.

All metal-conjugated antibodies in the panel were subjected to titration on pilot PBMC samples to determine the minimum amount of each antibody offering optimal resolution between positive and negative populations. Additional validation was performed by comparing cell populations obtained by CyTOF with assessment by flow cytometry. The Helios™ mass cytometer (Fluidigm, CA) was tuned and calibrated prior to each individual batch of acquisition of stained samples according to manufacturer’s recommendations to ensure consistent and accurate signal detection.

**Fig. 2:**
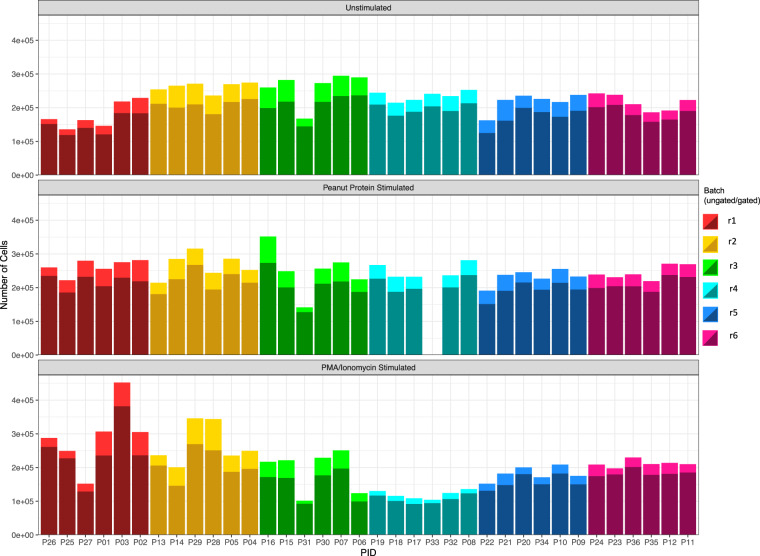
Number of cells per FCS file, stratified by stimulation condition. PBMCs from 36 infants underwent three different forms of stimulations; media alone (unstimulated, top), peanut-solution (allergen-specific stimulus, middle, no data available for participant P33), and PMA and ionomycin (non-specific stimulus, bottom). Each bar represents the total number of cells per sample, with different colors delineating the 6 batches. The darker shaded areas indicate the number of cells remaining after gating for live, single cells.

Potential batch effects were minimized for cell type frequencies and median expression analysis in the experimental design process. Samples within the three clinical groups were split across six batches. Meanwhile, for each participant, the three different stimulation conditions were generated within the same batch (Fig. 2). In the main study, the batch minimization of this design was illustrated by analyzing fold changes of frequencies of peanut-specific CD4 T cells after peanut stimulation over no stimulation.

Across the six batches and three different stimulations, the yield of live, single cells was checked and found to be comparatively consistent (Fig. 2). On average 16.85% (range: 8.74% - 27.25%, median: 16.05%) of events were excluded from the live, single cell gates. The average was 17.89% without stimulation, 16.53% after peanut stimulated, and 16.11% after stimulation with PMA/ionomycin.

## Supplementary information

Supplementary Figure S1

## Data Availability

Custom code was not used in the generation of any of the files and data that are available for public access.
