# Supplementary material for: Mass cytometry analysis of blood from peanut-sensitized tolerant and clinically allergic infants
Source: Sci Data. 2022 Dec 1;9:738. doi: 10.1038/s41597-022-01861-x (PMC9715645; doi:10.1038/s41597-022-01861-x)
Supplement: Supplementary file 1 — Supplementary Figure S1 [file 41597_2022_1861_MOESM1_ESM.pdf]

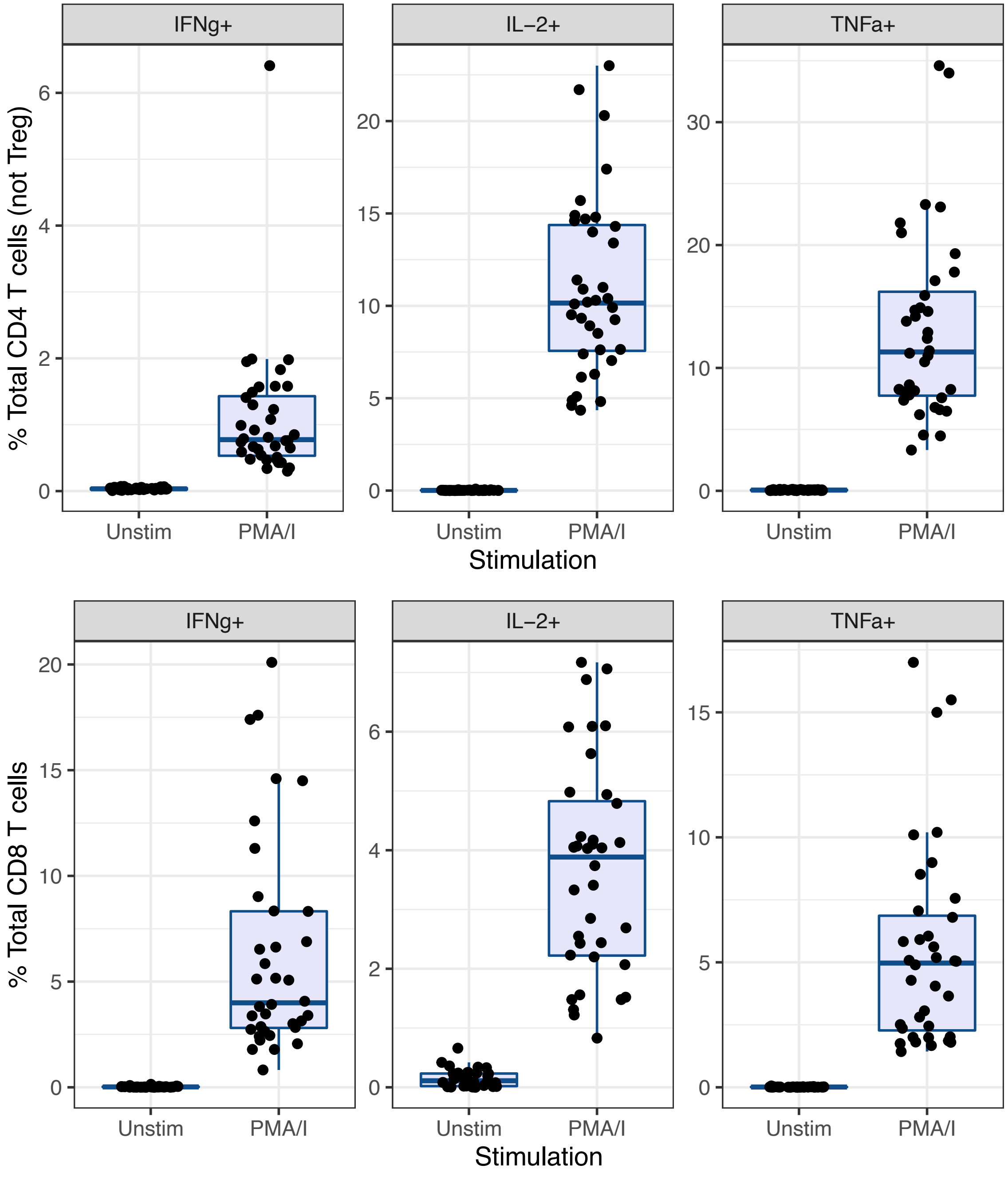

Supplementary Figure S1: Percentage of IFNγ+, IL-2+ and TNFα+ total CD4 T cells (excluding regulatory T cells) and total CD8 T cells. The percentages were significantly greater after stimulation with PMA and ionomycin (PMA/I) when compared to unstimulated (media alone) samples.
